# Unraveling the complexity of PD-L1 assays: a descriptive review of the methodology, scoring, and practical implications

**DOI:** 10.3389/fonc.2025.1581275

**Published:** 2025-12-03

**Authors:** Gilad W. Vainer, Sunil S. Badve, Pathmanathan Rajadurai, Fernando A. Soares, Giuseppe Viale, Reinhard Büttner, Shanthy Nuti, Jonathan Juco, Radha Krishnan, Ming-Sound Tsao

**Affiliations:** 1 Hadassah–Hebrew University Medical Center, Jerusalem, Israel; 2 Emory Winship Cancer Institute, Atlanta, GA, United States; 3 Monash University Malaysia, Selangor, Malaysia Subang Jaya Medical Centre, Subang Jaya, Malaysia; 4 Instituto de Anatomia Patológica, Rede D’Or São Luiz, São Paulo, Brazil; 5 Instituto de Pesquisa e Ensino D’Or, São Paulo, Brazil; 6 IEO, European Institute of Oncology IRCCS, Milan, Italy; 7 University of Cologne Medical Faculty, Cologne, Germany; 8 Merck & Co., Inc., Rahway, NJ, United States; 9 Princess Margaret Cancer Centre, University of Toronto, Toronto, ON, Canada

**Keywords:** immune checkpoint inhibitors, PD-L1 assays, PD-L1 expression, PD-L1 scoring method, regulatory approval

## Abstract

**Purpose:**

Over the years, immune checkpoint inhibitors targeting the programmed cell death protein 1 (PD-1) and programmed cell death ligand 1 (PD-L1) axes have substantially improved clinical outcomes for patients with various types of cancer and stages. As a result, PD-L1 is the most recognized biomarker used to guide the selection of patients for treatment with anti–PD-(L)1 therapy. To date, there are 4 regulatory agency-approved and commercially available immunohistochemistry assays used to quantify PD-L1 tumor expression, with each assay approved for use with a specific PD-(L)1 inhibitor. In this descriptive review, we concisely summarize the methodology and scoring methods of each assay, as well as some of the challenges associated with real-world use of these assay systems.

**Results:**

Each assay system is optimized for specific therapies, with its own anti-PD-L1 antibody, protocol, scoring, and interpretation guidelines. Although the methodologies of the 4 PD-L1 immunohistochemistry assay systems are similar, differences in their antibody clones, protocol conditions, instrumentation, and scoring methods limit assay interchangeability. The assays are also highly sensitive; slight deviations to the protocol can increase the risk of misclassifying the PD-(L)1 tumor status of patients. As a result, pathologists are faced with choosing which assay to perform with a limited tumor sample as well as with the challenges associated with the scoring methods and differences in regional regulatory approvals and infrastructure.

**Conclusion:**

While the 4 approved PD-L1 immunohistochemistry assays provide clinical value, we offer pathologists suggestions to reduce the challenges associated with PD-L1 testing based on assay systems.

## Introduction

Immune checkpoint inhibitors targeting programmed cell death receptor 1 (PD-1) and programmed cell death ligand 1 (PD-L1) have substantially improved clinical outcomes in multiple tumor types (1–9). As a result, PD-L1 is now one of the most widely used biomarkers to identify patients for treatment with immune checkpoint inhibitors (10). To date, there are 4 commercially available PD-L1 immunohistochemistry (IHC) assays that are approved for use in the United States (US Food & Drug Administration [FDA]), Europe (Conformité Européenne *in vitro* diagnostic [IVD] certification), and Japan (Pharmaceuticals and Medical Devices Agency [PMDA]) (11, 12): PD-L1 IHC 22C3 pharmDx (Agilent Technologies) (13), PD-L1 IHC 28–8 pharmDx (Agilent Technologies) (14), PD-L1 SP263 (Ventana/Roche) (15), and PD-L1 SP142 (Ventana/Roche) (16). These assays were codeveloped alongside a specific PD-(L)1 inhibitor in early-phase clinical trials (PD-L1 IHC 22C3 pharmDx: pembrolizumab [Merck Sharp & Dohme LLC, a subsidiary of Merck & Co. Inc., Rahway, NJ, USA]; PD-L1 IHC 28–8 pharmDx: nivolumab [Bristol Myers Squibb]; PD-L1 SP263 assay: durvalumab [AstraZeneca]; PD-L1 SP142 assay: atezolizumab [Genentech]), resulting in their approval as companion or complementary diagnostic assays (17, 18). Therefore, each diagnostic assay is optimized for specific therapies, with its own PD-(L)1 antibody, protocol, platform, and scoring and interpretation guidelines (19). Although each PD-L1 assay is well established with a PD-(L)1 inhibitor, differences in the assay systems restrict interchangeability and warrant closer examination.

Herein, we provide a concise descriptive review of all 4 commercially available PD-L1 IVD assay systems and their approved indications. We also offer suggestions for how pathologists can navigate challenges associated with the real-world use of these assay systems.

## Drug-diagnostic systems

**Figure 1:**
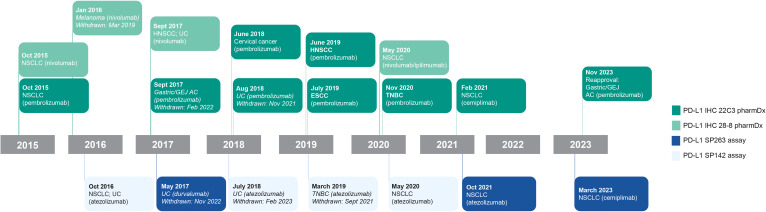
PD-L1 diagnostic assay FDA regulatory approvals. Bolded indications represent approval as a companion diagnostic. Non-bolded indications represent approval as a complementary diagnostic. Italicized text represents withdrawn indication. Approval status may vary by region; please refer to individual country labeling for more information. AC, adenocarcinoma; GEJ, gastroesophageal junction; HNSCC, head and neck squamous cell carcinoma; NSCLC, non–small cell lung cancer; PD-L1, programmed cell death ligand 1; TNBC, triple-negative breast cancer; UC, urothelial carcinoma.

The 4 currently approved and commercially available PD-L1 diagnostic assays are summarized in **Table 1** (13–16), and their regulatory US approval histories in **Figure 1**. Each PD-L1 diagnostic assay has been approved as a companion diagnostic or complementary assay (13–16).

**Table 1:** Summary of PD-L1 scoring methods.

Assay system	Scoring method				
	TPS	CPS	TC	IC	% PD-L1 expression in tumor cells
	PD-L1 IHC 22C3 pharmDx	PD-L1 IHC 22C3 pharmDx	PD-L1 SP263 assay PD-L1 SP142 assay	PD-L1 SP142 assay	PD-L1 IHC 28–8 pharmDx
PD-L1 expression on TCs	✓	✓	✓		✓
PD-L1 expression on ICs		✓		✓	
Normalized by number of TCs	✓	✓	✓		✓
Normalized by cross-sectional area				✓	

CPS, combined positive score; IC, immune cell; IHC, immunohistochemistry; PD-L1, programmed cell death ligand 1; TC, tumor cell; TPS, tumor proportion score.

### PD-L1 IHC 22C3 pharmDx

PD-L1 IHC 22C3 pharmDx (13) was developed to detect PD-L1 expression and facilitate the safe and effective use of the PD-1 inhibitor pembrolizumab in patients with non–small cell lung cancer (NSCLC) (20). This assay was first approved as a companion diagnostic by the FDA in 2015 (21, 22), followed by approvals for the same indication in Europe and Japan in 2016. PD-L1 IHC 22C3 pharmDx has since been approved as a companion diagnostic for use with pembrolizumab in other tumor types, including cervical cancer, esophageal squamous cell carcinoma (ESCC), gastric or gastroesophageal junction (GEJ) adenocarcinoma, head and neck squamous cell carcinoma (HNSCC), triple-negative breast cancer (TNBC), and urothelial carcinoma (UC; FDA label subsequently updated to remove PD-L1 tumor expression requirement) (13). In 2021, the assay was approved by the FDA for use with another PD-1 inhibitor, cemiplimab-rwlc, in patients with NSCLC (23).

### PD-L1 IHC 28–8 pharmDx

PD-L1 IHC 28–8 pharmDx (14) was developed to detect PD-L1 expression and facilitate the safe and effective use of the PD-1 inhibitor nivolumab in patients with NSCLC (24). This assay was first granted FDA approval in 2015 as a complementary diagnostic, followed by European approval in 2016 (25). Since then, complementary diagnostic approval by the FDA has been extended to patients with HNSCC, melanoma (subsequently withdrawn), and UC (26). In 2020, the assay was approved by the FDA as a companion diagnostic for use with nivolumab plus ipilimumab in NSCLC (27); this approval was also granted in Japan and Europe (28, 29), both of which also approved its use in ESCC (29, 30), HNSCC (29, 31), gastric cancer (29, 32, 33), and melanoma (28, 29). The assay is also approved for use with nivolumab in Europe for muscle-invasive UC, GEJ adenocarcinoma and esophageal adenocarcinoma (34).

### PD-L1 SP263 assay

The PD-L1 SP263 assay (15) was developed to detect PD-L1 expression for use with the PD-L1 inhibitor durvalumab in UC (35). The FDA first approved this assay as a complementary diagnostic for use with durvalumab in UC in 2017 (subsequently withdrawn) (35), followed by approvals as a companion diagnostic for use with atezolizumab and cemiplimab-rwlc in NSCLC (35). In addition to these US approvals, the PD-L1 SP263 assay was approved in Japan as a companion diagnostic for use with atezolizumab for NSCLC (36) and in Europe as a companion diagnostic for use with either atezolizumab or cemiplimab-rwlc in NSCLC (37, 38).

### PD-L1 SP142 assay

The PD-L1 SP142 assay (16) was developed to detect PD-L1 expression for companion use with the PD-L1 inhibitor atezolizumab in NSCLC and UC (39). This assay was first approved by the FDA as a complementary diagnostic in 2016 (40). This was followed by US approval of the assay as a companion diagnostic for use with atezolizumab for NSCLC (41), TNBC (subsequently withdrawn), and UC (subsequently withdrawn). The assay has also been approved as a companion diagnostic for use with atezolizumab in Europe (NSCLC, TNBC, and UC) and Japan (NSCLC and TNBC) (36, 42, 43).

## Protocols and chemistry

**Figure 2:**
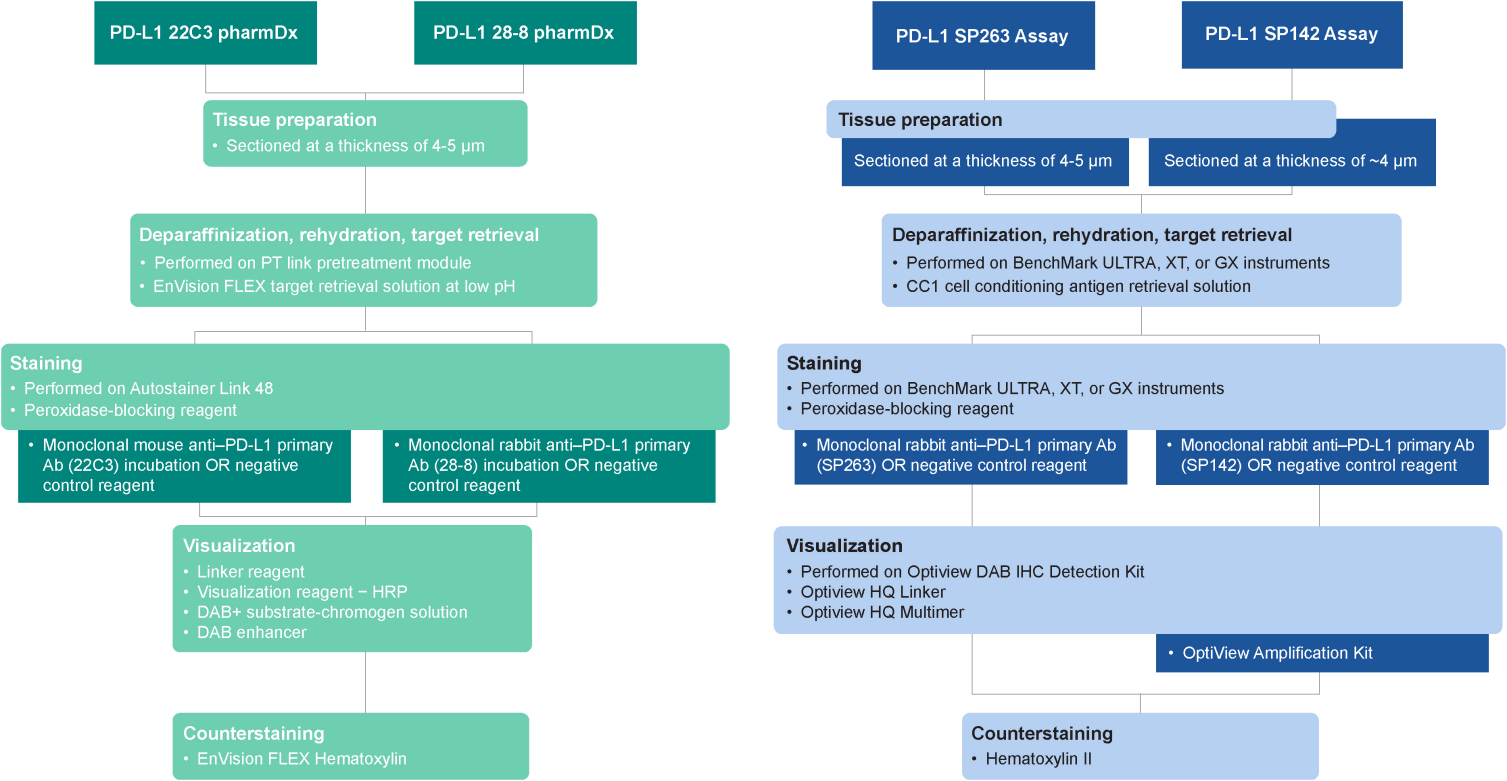
Summary of protocols for commercially available PD-L1 diagnostic assays. Ab, antibody; CC1, cell conditioning 1; DAB, 3,3’-diaminobenzidine; HRP; horseradish peroxidase; IHC, immunohistochemistry; PD-L1, programmed cell death ligand 1.

Recommended IHC staining protocols of the 4 commercially available PD-L1 diagnostic assays are summarized in **Figure 2**.

### PD-L1 IHC 22C3 pharmDx

PD-L1 IHC 22C3 pharmDx (13, 44) is optimized for use with the PT Link Pretreatment Module (Agilent Technologies, Carpinteria, CA), Autostainer Link 48 automated staining platform (Agilent Technologies), and Dako Omnis (Agilent Technologies), and visualized with the EnVision FLEX system (Agilent Technologies). Briefly, formalin-fixed paraffin-embedded (FFPE) tissue samples are fixed in 10% neutral buffered formalin (NBF) for 12–72 hours, embedded in paraffin, cut into 4- to 5-µm sections, and mounted on positively charged microscope slides. Prepared tissue sections are incubated with an anti–PD-L1 22C3 mouse monoclonal primary antibody followed by a Linker antibody specific to the host species of the primary antibody. This step is followed by incubation with a secondary antibody conjugated to a horseradish peroxidase (HRP) chromogen. The sample may then be counterstained and viewed under a light microscope for interpretation of the results. Preparation of a second slide stained with the negative control [immunoglobulin (Ig)] reagent (NCR) can help assess background staining, and positive control slides containing 2 FFPE human cell lines are provided to validate staining runs.

### PD-L1 IHC 28–8 pharmDx

Similar to PD-L1 IHC 22C3 pharmDx, PD-L1 IHC 28–8 pharmDx (14) is optimized for use with the PT Link Pretreatment Module and Autostainer Link 48 automated staining platform and visualized with the EnVision FLEX system. FFPE tissue samples are fixed in 10% NBF for 24–48 hours, embedded in paraffin, cut into 4- to 5-µm sections, and mounted on positively charged microscope slides. The IHC staining procedure uses an anti–PD-L1 28–8 rabbit monoclonal primary antibody and follows a protocol very similar to PD-L1 IHC 22C3 pharmDx.

### PD-L1 SP263 assay

The PD-L1 SP263 assay (15) is optimized for use with the OptiView DAB IHC Detection Kit on the BenchMark ULTRA automated staining platform (Roche Diagnostics, Indianapolis, IN). FFPE tissue samples are fixed in 10% NBF for 6–72 hours, embedded in paraffin, cut into 4- to 5-µm sections, and mounted on microscope slides. Prepared tissue sections are incubated with an anti–PD-L1 SP263 rabbit monoclonal primary antibody followed by incubation with the OptiView HQ Linker (Roche Diagnostics) for 8 minutes, and then a multimer for an additional 8 minutes. The sample may then be counterstained, post-counterstained, and viewed under a light microscope. Preparation of a second slide stained with the NCR (rabbit monoclonal negative control Ig) can help assess background staining and a positive control slide of qualified normal human term placental tissue can be prepared to validate staining runs.

### PD-L1 SP142 assay

**Figure 3:**
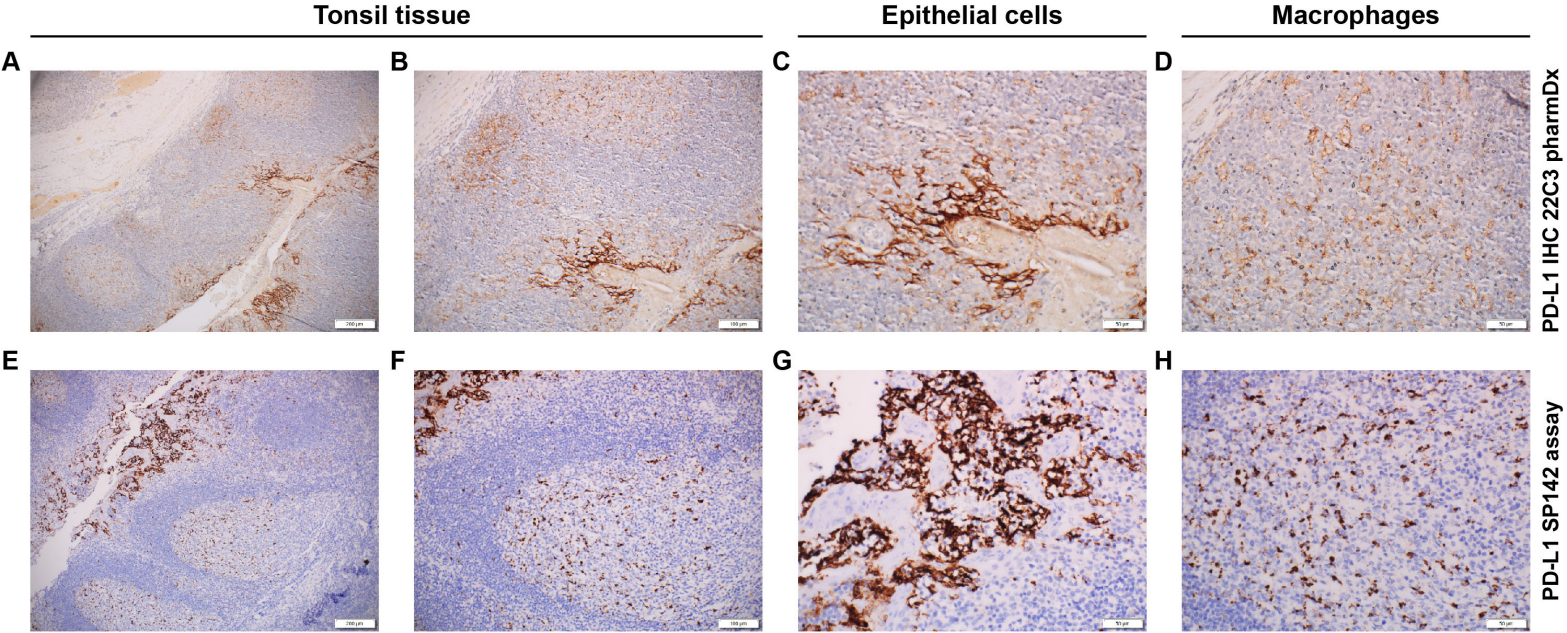
Immunohistochemical staining pattern of PD-L1 using different assay systems. **(A–D)** PD-L1 IHC 22C3 pharmDx staining. **(A)** Positive epithelial cells (right) with low background noise. **(B)** Higher magnification of the positive epithelial cells. **(C)** Positive epithelial cells of tonsil tissue (brown pigment). **(D)** Macrophages of the tonsil tissue. **(E–H)** PD-L1 SP142 assay staining. **(E)** Positive epithelial cells (left) with high background noise. **(F)** Higher magnification of the positive epithelial cells. **(G)** Positive epithelial cell of the tonsil tissue (brown pigment). **(H)** Macrophages of the tonsil tissue. Magnification: **(A, E)**, ×10; **(B, F)**, ×20; **(C, D, G, H)**, ×40.

The PD-L1 SP142 assay (16) is optimized for use with the OptiView DAB IHC Detection Kit and OptiView Amplification Kit on BenchMark ULTRA automated staining platform. FFPE tissue samples are fixed in 10% NBF for 6–72 hours, embedded in paraffin, cut into 4-μm sections, and mounted on microscope slides. Prepared tissue sections are incubated with an anti–PD-L1 SP142 rabbit monoclonal primary antibody, followed by incubation with the OptiView HQ Linker for 8 minutes and then a multimer for an additional 8 minutes. Importantly, amplification with a hapten amplifier (for 8 minutes) and amplification multimer (for 8 minutes) is performed; this produces a dark, granular staining pattern not usually seen in the other 3 assays (**Figure 3**). The sample is counterstained, post-counterstained, and viewed under a light microscope. Preparation of a second slide stained with the NCR (rabbit monoclonal negative control Ig) can help assess background staining, and a positive control slide of qualified benign human tonsil tissue can be prepared to validate staining runs.

## Scoring methods

The scoring algorithms used with the 4 commercially available PD-L1 assays estimate the percentage of PD-L1–positive tumor cells (TCs), immune cells (ICs), or both (**Table 1**). The tumor proportion score (TPS), percentage PD-L1 expression in tumor cells, and TC staining assessment all reflect the percentage of TCs that express PD-L1. TPS is defined as the percentage of viable TCs showing partial or complete PD-L1 membrane staining at any intensity (45). The percentage TC score is similar to the TPS and is defined as the percentage of evaluable TCs exhibiting partial linear or complete circumferential plasma membrane PD-L1 staining at any intensity (46), whereas TC staining assessment is defined as the presence of discernible PD-L1 membrane staining of any intensity in TCs (16). In contrast, the IC staining assessment is the only scoring system normalized by the cross-sectional area of the section defined as the percentage of tumor area occupied by PD-L1–expressing tumor-infiltrating ICs (lymphocytes, plasma cells, macrophages, and granulocytes) of any intensity (16). Finally, the combined positive score (CPS) is defined as the number of PD-L1-staining cells (TCs, lymphocytes, and macrophages) divided by the total number of viable TCs, multiplied by 100 (and capped at 100) (45).

Scoring methods and their cutoffs to define PD-L1 expression vary considerably by type of diagnostic assay, tumor type, and treatment strategy (**Table 2**). For example, all 4 PD-L1 assays received FDA approval for use as companion diagnostics with different first-line treatment strategies in NSCLC, using various cutoffs to define PD-L1 expression (47). Pembrolizumab monotherapy was the first PD-1 inhibitor therapy approved for the first-line treatment of patients with metastatic NSCLC, specifically those whose tumors have high PD-L1 expression (TPS ≥50%), assessed per PD-L1 IHC 22C3 pharmDx (48). This approval was later expanded to include pembrolizumab for first-line treatment of patients with advanced or metastatic NSCLC with PD-L1 expression of TPS ≥1% (48) and cemiplimab-rwlc monotherapy for first-line treatment of metastatic NSCLC with TPS ≥50% (49). PD-L1 IHC 28–8 pharmDx also received FDA approval for use in identifying candidates for first-line therapy with the combination of nivolumab plus ipilimumab for metastatic NSCLC with a percentage TC expression of ≥1% (27). The PD-L1 SP142 assay was also approved for use with atezolizumab as the first-line treatment of metastatic NSCLC (TC ≥50% or IC ≥10%) (41).

**Table 2:** Commercially available PD-L1 diagnostic assays.

PD-L1 diagnostic assay	Antibody clone	Platform	Detection system	PD-(L)1 inhibitor	Clinical scoring	Approved PD-L1–positive tumors
PD-L1 IHC 22C3 pharmDx (Dako/Agilent) (13)	22C3	Autostainer Link 48	EnVision FLEX	Pembrolizumab	TPS	• NSCLC (≥1%)
					CPS	• Cervical cancer (≥1) • Esophageal cancer (≥10) • Gastric or GEJ adenocarcinoma (≥1) • HNSCC (≥1) • TNBC (≥10)
				Cemiplimab	TPS	• NSCLC (≥50%)
PD-L1 IHC 28–8 pharmDx (Dako/Agilent) (14)	28-8	Autostainer Link 48	EnVision FLEX	Nivolumab	% PD-L1 expression in tumor cells	• HNSCC (≥1%) • Non-squamous NSCLC (≥1%, ≥5%, or ≥10%) • NSCLC (≥1%) • UC (≥1%)
PD-L1 SP263 assay (Ventana) (15)	SP263	BenchMark ULTRA	OptiView	Atezolizumab	TC	• NSCLC (≥1%)
				Cemiplimab	TC	• NSCLC (≥50%)
PD-L1 SP142 assay (Ventana) (16)	SP142	BenchMark ULTRA	OptiView (detection and amplification)	Atezolizumab	TC/IC	• NSCLC (≥50% TC or ≥10% IC) • TNBC (IC ≥1%) • UC (IC ≥5%)

CPS, combined positive score; ESCC, esophageal squamous cell carcinoma; GEJ, gastroesophageal junction; HNSCC, head and neck squamous cell carcinoma; IC, immune cell; IHC, immunohistochemistry; NSCLC, non–small cell lung cancer; PD-1, programmed cell death protein 1; PD-L1, programmed cell death ligand 1; TC, tumor cell; TNBC, triple-negative breast cancer; TPS, tumor proportion score; UC, urothelial carcinoma.

Approval status based on the FDA. Approval status may vary by region; please refer to individual country labeling for more information.

## Practical considerations and challenges

The availability of multiple PD-L1 IHC assay systems and treatment strategies make it challenging to identify patients likely to respond to specific therapies. The Blueprint PD-L1 IHC Assay Comparison Project performed analytical and clinical comparisons of the 4 assay systems in NSCLC alone (50, 51). During phase 1, analytical comparison indicated that 3 of the 4 assays (PD-L1 22C3 pharmDx, PD-L1 28–8 pharmDx, and PD-L1 SP263) resulted in similar proportions of PD-L1–stained TCs; however, fewer PD-L1–stained TCs overall were observed for the PD-L1 SP142 assay (50). Although all 4 assays detected ICs, greater variability of IC PD-L1 staining was observed. Additionally, the PD-L1 SP142 assay resulted in staining that was more punctate and discontinuous, which may be a reflection of its unique additional amplification step (steep reaction curve) of this assay (50). Comparison of clinical performance during phase 1 demonstrated that interchanging validated cutoff thresholds between assays reduced the rate of agreement compared with the reference standard (defined as the validated cutoff and assay combination) (50). Therefore, there is potential for “misclassification” of PD-L1 status of some tumors if assays and validated cutoffs are interchanged (50).

Analytical validation of the 4 assay systems during phase 2 confirmed the findings observed during phase 1 and demonstrated strong reliability among pathologists in TC PD-L1 scoring and poor reliability in IC PD-L1 scoring (51). Notably, Tsao et al. stated that the use of different anti–PD-L1 monoclonal primary antibody clones with potential variances in epitope binding may result in distinct staining properties, thus prohibiting their interchangeability in the clinical setting (51). In support of this, a detailed mapping analysis of PD-L1 antibody clones showed that the discordance of staining between the SP142 and the SP263 assays, despite targeting an identical epitope, was likely due to subtle differences in assay protocol design rather than antibody specificity (47). Additionally, staining intensity and patterns varied by tumor type for each antibody clone (46). Notably, in selected lung cancer cases, therapy-relevant differences in PD-L1 tumor cell expression were observed depending on the antibody used (46). Finally, it is important to note that greater sensitivity of a PD-L1 IHC assay system does not imply a higher predictive value for response to drug treatment (52).

From the regulatory perspective, a companion diagnostic assay is a medical device, often an IVD, that provides information essential for the safe and effective use of a corresponding therapeutic product, while a complementary diagnostic assay identifies a biomarker-defined subset of patients who are more likely to respond to a drug, providing clinically meaningful information that aids in risk-benefit assessments for individual patients (53). In comparison with companion diagnostic assays, complementary diagnostic assays are not prerequisites for receiving a treatment (53). As a result, clinicians may be less inclined to order testing with PD-L1 complementary diagnostics, particularly in jurisdictions for which there is insufficient reimbursement and the costs are a concern. While the FDA has approved use of the 4 assay systems as complementary diagnostics in some tumor types (eg, PD-L1 IHC 28–8 pharmDx with nivolumab for HNSCC and UC), the European Medicines Agency does not recognize complementary diagnostic assays for use with immune checkpoint inhibitors.

From the infrastructure perspective, pathology laboratories may not own the Autostainer Link 48 or the BenchMark ULTRA staining platform and may therefore use another IHC staining platform or a laboratory-developed test (LDT) to assess PD-L1 expression (54). LDTs are a type of diagnostic test for which the protocol was designed and developed in the test laboratory; LDTs may also include previously approved tests that have been altered in some way (55). These assays are generally developed out of necessity due to the lack of instruments to perform the commercially available assays according to their required specifications (56). However, technical validation of LDTs must be undertaken before an assay can be considered similar to a clinically validated companion diagnostic (57). Recently, the College of American Pathologists (CAP) published updated guidelines on the analytic validation of IHC assays (58). The CAP recommends that all LDTs must be analytically validated before reporting results on patient tissue, and laboratories should achieve ≥90% overall concordance between the LDT and the comparator assay or expected results (58). Furthermore, analytic validation can be established for predictive LDTs by testing a minimum of 20 positive and negative tissue samples each, and each assay-scoring system should be separately validated with a minimum of 20 positive and negative tissue specimens each (58). Despite these guidelines, concerns about the use of LDTs still exist, including the complexity of LDT validation given variability in IHC assay analytic validation practices, the lack of transparency regarding methodology that is often not publicly available, the lack of external review, and the limited oversight/governance. Recently, the FDA announced that IVDs, including those manufactured by a laboratory, are included under the Federal Food, Drug and Cosmetic Act (59). The revised policy provides greater oversight to ensure the safety and effectiveness of LDTs in the US (59).

It is well known that changes in protocols, however minor, may dramatically change the IHC staining pattern. Thus, it is unsurprising that studies have shown that minor changes in IHC protocols can have a substantial impact on PD-L1–staining patterns and intensity (60). In a study evaluating the reliability and reproducibility of the 22C3 antibody with the Ventana BenchMark XT platform (Roche Diagnostics), decreasing the antibody incubation time from 32 to 16 minutes resulted in the germinal center of macrophages being mostly negative (61). Substantial variability in staining may also result from differences in the processing and embedding of tissue (**Table 3**) (62). Therefore, in addition to cell line controls, the selection and use of positive and negative control tissue from specimens of the same tumor type as the patient specimen in each staining run is recommended (63). However, because this practice is hard to manage clinically, positive control tissue with a complete dynamic representation of the staining can be used. For example, reactive human tonsil, normally shows strong positivity in the epithelium and light positivity of the germinal center macrophages (so called “tangible body macrophages”) (61). This control demonstrates easily the dynamic range of the PD-L1 staining and can be incorporated on each slide. We do not recommend using placenta as a positive internal control. Placenta stains very strongly for PD-L1 by IHC, and because the tissue lacks the full dynamic range of the staining, the interpreting pathologist might miss a sensitivity drift in the moderate- to low-expressing cells. Laboratories should consider including the interpretation of the internal-positive control of the patient’s tissue (macrophages, lymphocytes, etc) in the patient’s report. This is particularly important with low levels or negative PD-L1–expressing biopsies, to identify drifts in staining due to pre-analytical conditions. The identification and reporting of suboptimal internal control positive and negative cells should be considered, as this provides the treating oncologist more information about the validity and adequacy of the score.

**Table 3:** Limitations and special considerations of the PD-L1 diagnostic assays.

PD-L1 diagnostic assay	Limitations and considerations	Special considerations
PD-L1 IHC 22C3 pharmDx (Dako/Agilent) (1)	• It is recommended to stain specimens within recommended cut section storage limits to prevent antigen degradation and false negatives • Target retrieval pre-treatment is required for optimal PD-L1 staining in NBF-fixed and paraffin-embedded tissue • Do not substitute reagents from different lots or other manufacturers, except EnVision FLEX Target Retrieval Solution, low pH (50x) • Control cell lines validate staining runs only and should not guide patient tissue scoring • Use on tissue prepared with fixatives other than 10% NBF has not been validated • It is not validated for use on fine needle aspirates and decalcified tissues • Laboratories should monitor the pH of 1x EnVision FLEX Target Retrieval Solution; low pH (pH 5.9) for pre-treatment of ESCC specimens may affect PD-L1 staining • Studies evaluating up to 3 uses of 1x EnVision FLEX Target Retrieval Solution, low pH in esophageal cancer failed to meet acceptance criteria for PD-L1 assessment; therefore, re-use is not recommended for ESCC specimens • For PD-L1 assessment in gastric or GEJ adenocarcinoma endoscopic biopsies, at least 3–5 biopsies are recommended	• Clinicians should exercise caution interpreting results at the CPS ≥20 cutoff, as PD-L1 IHC 22C3 pharmDx did not meet acceptance criteria for positive percentage agreement and overall percentage agreement in reproducibility studies on HNSCC specimens; however, all criteria were met at the CPS ≥1 cutoff
PD-L1 IHC 28–8 pharmDx (Dako/Agilent) (2)	• It is recommended to stain specimens within recommended cut section storage limits to prevent antigen degradation and false negatives • Target retrieval pre-treatment is required for optimal PD-L1 staining in NBF-fixed and paraffin-embedded tissue • Do not substitute reagents from different lots or other manufacturers, except EnVision FLEX Target Retrieval Solution, low pH (50x) • Control cell lines validate only staining runs and should not guide patient tissue scoring • Use on tissue prepared with fixatives other than 10% NBF has not been validated • Excisional, incisional, punch, or core needle biopsies are acceptable sample types; fine needle aspirates or other cytology specimens are insufficient for biomarker analysis	
PD-L1 SP263 assay (Ventana) (3)	• This was for the BenchMark ULTRA instruments with the OptiView DAB IHC Detection Kit and is not approved for use with any other detection method or instruments • A patient specimen slide should be stained with rabbit monoclonal negative control Ig; other negative controls are unsuitable • It is not validated for use with cytology samples or decalcified bone specimens • Cut slides should be desiccated and stored at room temperature; laboratories should validate slide stability beyond 45 days due to environmental impact on antigen stability	
PD-L1 SP142 assay (Ventana) (4)	• This was for the BenchMark ULTRA instrument with the OptiView DAB IHC Detection Kit and the OptiView Amplification Kit and is not approved for use with any other detection method or instruments • A patient specimen slide should be stained with rabbit monoclonal negative control Ig; other negative controls are unsuitable • The antibody is stable up to 8 days at 30 °C; performance beyond this is unestablished • It is not validated for use with cytology samples or decalcified bone specimens • Patient tissue should be stained within 2 months of sectioning from the tissue block for NSCLC and tonsil tissue; staining deteriorates when stored longer than 2 months at room temperature • It is recommended to fix samples in 10% NBF for 6–72 hours; other fixation times or fixatives (eg, AFA, Prefer, alcohol-based fixative) may cause false negatives by reducing PD-L1 staining • Artifacts such as DAB spots, blank spots, DAB dots, or speckling that affect interpretation may require repeat staining; always compare with negative control slides to confirm acceptable background • Occasional DAB dots have been observed in benign human tonsil control, cerebellum, and testicular tissue, and focal nuclear staining has been observed in normal pancreatic (acinar cells) and hypophyseal tissue; however, nuclear staining is not included when scoring	

AFA, alcohol-formalin-acetic acid; CPS, combined positive score; DAB, 3,3’-diaminobenzidine; ESCC, esophageal squamous cell carcinoma; GEJ, gastroesophageal junction; Ig, immunoglobulin; IHC, immunohistochemistry; NBF, neutral buffered formalin; NSCLC, non–small cell lung cancer; PD-L1, programmed cell death ligand 1.

Method of collection (by core biopsy or needle aspiration), tissue type, and tissue availability may pose challenges when evaluating PD-L1 status (64). For example, PD-L1 status is a poor predictor of treatment outcomes associated with immunotherapy in patients whose samples were obtained by needle aspiration (exfoliative cytology samples, pleural fluids especially; these occasionally are the only samples available) (64). Moreover, studies comparing core biopsy with surgical resection samples indicate that caution should be taken for specific biopsy specimens to evaluate PD-L1 status. For instance, immunostaining of decalcified bone biopsies and the impact of different decalcification solutions on antigen expression have not been validated, and additional sampling of the tumor from soft tissue may be required to minimize the risk of tumor misclassification (65–67). In contrast, some studies show comparable PD-L1 expression between cytology specimens and surgical resections (68, 69). Lau et al. showed that PD-L1 assessment on cytology specimens was comparable to histology and predicted similar treatment responses with single-agent immune checkpoint inhibitors in metastatic NSCLC (70). Furthermore, although there is generally good concordance in PD-L1 expression between primary and metastatic tumor samples (35, 71–73), discordance in PD-L1 expression among the origin of tissue samples can occur and should be considered (74–77). Some studies reported higher PD-L1 expression in metastatic sites in patients with breast cancer, lung cancer, or melanoma (74–77). Notably, in patients with TNBC, immunostaining with PD-L1 SP142 assay showed a lower percentage of PD-L1–positive cells in liver metastases compared with other metastatic sites (78). If tissue availability is limited, prioritization of PD-L1 IHC over other biomarkers may be required and is dependent on the tumor type.

Interpreting the results of IHC staining relies on the standard IHC assay approach of morphological evaluation (79). Assessment of PD-L1 expression by CPS is based on visual estimation of the positive expression of PD-L1 in tumor and immune cells. In samples with sparse cellularity or tumor content, where PD-L1 staining is close to the cutoff, pathologists may count tumor cells to arrive at an accurate estimation of the CPS cutoff (79). Because TPS requires visual estimation of the area covered by PD-L1–positive TCs, scoring may be subject to interobserver variability due to difficulties in estimating heterogeneous cell populations with intermixed areas of PD-L1–positive and PD-L1–negative tumor regions (79). Notably, complexities exist across tumor types, which may complicate assessment of PD-L1 expression. In HNSCC, when scoring PD-L1 expression, tumor-associated immune cells should be differentiated from chronic inflammation, and PD-L1–positive carcinoma *in situ* or dysplasia should be excluded when assessing invasive cancer (80). In TNBC, PD-L1 staining of neutrophils, eosinophils, and plasma cells is not common but, when present, may be difficult to exclude from scoring using CPS (62). When scoring gastric cancer samples stained by PD-L1 IHC 22C3 pharmDx, it is important to delineate immune cells from diffuse infiltrative signet ring cells to ensure accurate calculation of CPS.

Pathologist training on the use and interpretation of PD-L1 IHC assays may improve consistency and quality in the assessment of PD-L1 status (81, 82). For example, pathologists who underwent specific reader training to score PD-L1 using TPS or CPS with PD-L1 IHC 22C3 pharmDx showed high overall interobserver agreement across several tumor types (83, 84); interobserver agreement was ≥87.3% for TPS (83) and ≥86.5% for CPS (82). An online training tool for the PD-L1 SP263 assay and the PD-L1 IHC 22C3 pharmDx also resulted in high inter-reader concordance among pathologists for PD-L1 TC scoring (84). External proficiency surveys are also of value to assess practice patterns of PD-L1 IHC assay systems and identify issues and disparities (85). In the Nordic immunohistochemical Quality Control (NordiQC) external quality assessment (EQA) program for IHC, cumulative data for PD-L1 IC score revealed a mean pass rate of 88% (range, 78%-93%) for the PD-L1 SP142 assay, whereas other PD-L1 companion diagnostic assays (PD-L1 22C3 pharmDx, PD-L1 28–8 pharmDx, and PD-L1 SP263 assay) and LDTs provided a mean pass rate of 20% (range, 0%-74%) (85). A global survey of pathologists conducted by the International Association for the Study of Lung Cancer pathology committee highlighted marked heterogeneity in PD-L1 testing across regions and laboratories with regard to antibody clones, IHC assays, samples, turnaround times, and quality assurance measures (86).

## Discussion

Despite the availability of 4 approved PD-L1 assays, inter-laboratory differences in evaluation remain a challenge. To improve this, it is recommended that pathologists utilize the available online training tools prior to validating these assays in their laboratories. Additionally, laboratories that perform PD-L1 IHC staining must continuously participate in EQA programs (eg, CAP, NordiQC, United Kingdom National External Quality Assessment Service, and European Society of Pathology) (87–90). Participating laboratories should select an EQA program that will inform the laboratory on the accuracy of the PD-L1 IHC assay protocol and the pathologists’ readout based on assessment by a central expert panel. However, participation in EQA programs is not a substitute for clinical and technical validation.

Laboratories can assess assay system proficiency by comparing their observed PD-L1 positivity and negativity rates across reported tumor types against benchmark data from tumor type–specific clinical trials. This comparison helps identify pre-analytical, analytical, and post-analytical processes, serving as a simple and cost-effective best practice for scoring PD-L1 using each assay system.

Complexities associated with the interpretation and scoring of PD-L1 IHC results support the rationale for developing computer-assisted PD-L1 assessment tools (eg, digital pathology, deep learning, artificial intelligence [AI]) to overcome challenges associated with manual evaluation of PD-L1 status. Recently, an AI tool was used to evaluate PD-L1 IC expression in breast cancer, substantially improving accuracy and concordance in the interpretation of PD-L1 status (91). AI-assisted methods have also been investigated in other tumor types, including NSCLC and HNSCC (92–95). However, adequate testing, validation, and training using an AI approach are required to ensure that the AI algorithm is robust, reproducible, and acceptable to support pathologists as a tool for assessment of PD-L1 expression.

In conclusion, pathologists experience multiple challenges associated with PD-L1 testing using the 4 commercially available PD-L1 IHC assay systems. Studies have shown that some of these assays are comparable but not interchangeable, highlighting differences in the entire system of detection. As a result, deviation from the protocol of a validated PD-L1 IHC assay system may increase the risk of misclassifying PD-L1 status. In addition, regulatory variability further challenges the consistent use of the PD-L1 assays, with regional differences in approvals of companion versus complementary diagnostics impacting clinical adoption. Moreover, many laboratories face constraints due to lack of approved staining platforms and infrastructure limitations, necessitating reliance on rigorously validated LDTs. While both commercially available assays and LDTs are of clinical value, pathologists should be mindful that all systems have their limitations. Therefore, laboratories and pathologists are encouraged to use well-validated, peer-reviewed LDT protocols, followed by validation of the LDTs, and to further assess, if possible, commercially available systems in different clinical settings.
